# Chronic renal comorbidities in pyoderma gangrenosum: a retrospective cohort study

**DOI:** 10.1007/s12026-021-09187-3

**Published:** 2021-04-22

**Authors:** Khalaf Kridin, Arieh Solomon, Rimma Laufer Britva, Dana Tzur Bitan, Arnon D. Cohen

**Affiliations:** 1 Lübeck Institute of Experimental Dermatology, University of Lübeck, Ratzeburger Allee 160, 23562 Lübeck, Germany; 2 Azrieli Faculty of Medicine, Bar-Ilan University, Safed, Israel; 3 Clalit Health Services, Tel-Aviv, Israel; 4 Technion, Israel Institute of Technology, Haifa, Israel; 5 Department of Behavioral Sciences, Ariel University, Ariel, Israel; 6 Siaal Research Center for Family Medicine and Primary Care, Faculty of Health Sciences, Ben-Gurion University of the Negev, Beer Sheva, Israel

**Keywords:** Pyoderma gangrenosum, Renal comorbidities, Chronic renal failure, Dialysis, Kidney transplantation, Other kidney diseases

## Abstract

The coexistence of pyoderma gangrenosum (PG) and chronic renal comorbidities has been reported anecdotally. We aimed to assess the bidirectional association between PG and the following chronic renal comorbidities: chronic renal failure (CRF), dialysis, kidney transplantation (KT), and other kidney diseases (OKD). That is to evaluate (i) the risk of the aforementioned diseases among patients with PG (ii) and the odds of PG after a diagnosis of renal comorbidities. A population-based retrospective cohort study was conducted comparing PG patients (*n*=302) with age-, sex-, and ethnicity-matched control subjects (*n*=1497) with regard to incident cases of renal comorbidities. A case-control design was additionally adopted to estimate the odds of PG in those with a preexisting history of renal comorbidities. Adjusted hazard ratios (HRs) and adjusted odds ratios (ORs) were estimated by Cox regression and logistic regression, respectively. Patients with PG demonstrated an increased risk of CRF (adjusted HR, 3.68; 95% CI, 2.72–5.97), dialysis (adjusted HR, 27.79; 95% CI, 3.24–238.14), and OKD (adjusted HR, 2.71; 95% CI, 1.55–4.74). In addition, the odds of PG were increased after the diagnosis of CRF (adjusted OR, 2.34; 95% CI, 1.33–4.11), KT (adjusted OR, 5.03; 95% CI, 1.01–25.12), and OKD (adjusted OR, 1.69; 95% CI, 1.04–2.74). Patients with a dual diagnosis of PG and renal diseases presented with PG at an older age and had a higher prevalence of comorbid conditions. In conclusion, a bidirectional association exists between PG and chronic renal conditions. Awareness of this comorbidity may be of benefit for physicians managing patients with PG.

**Supplementary Information:**

The online version contains supplementary material available at 10.1007/s12026-021-09187-3.

## Introduction

Pyoderma gangrenosum (PG) is a neutrophilic inflammatory skin disease that typically progresses over days from a painful nodule, plaque, or pustule to ulceration with undermined, violaceous borders and a surrounding zone of erythema [1]. Different variants of PG exist, such as ulcerative, bullous, pustular, vegetative, peristomal, and post-surgical [2]. Based on numerous retrospective studies, the annual incidence of PG was estimated at 3 to 10 cases per million [2, 3].

PG is associated with underlying systemic conditions in 56.8% of patients, with inflammatory bowel disease, inflammatory arthritis, and hematological malignancies being the most frequently encountered underlying comorbidities [4]. A better characterization of underlying diseases is of substantial importance as the type and severity of these comorbidities bear a prognostic value for PG [5, 6]. PG is a prototype of neutrophilic dermatoses, a polymorphous group of noncontagious dermatological disorders characterized by non-cutaneous findings including systemic inflammation, non-specific inflammatory findings (e.g., myalgia, fever, and joint pain), and sterile neutrophilic infiltrates found in organs other than skin [7, 8]. The visceral involvement may support the systemic nature of PG among other neutrophilic dermatoses [8].

While the most common extracutaneous manifestation of PG is pulmonary conditions, some case reports described its association with chronic renal comorbidities [7]. However, the association of PG with renal comorbidities is yet to be systematically investigated. The aim of this real-world data analysis was to evaluate the bidirectional association between PG and renal comorbidities.

## Methods

### Study design and database

The current study was performed to investigate the bidirectional association between PG and renal comorbidities. To outline the risk of developing renal diseases during the course of PG, a retrospective cohort study design was adopted, in which patients with PG were followed to estimate the incidence of renal diseases. To evaluate the risk of having PG in individuals with a preceding history of renal diseases, a case-control study design was implemented to disclose the prevalence of preexisting renal diseases (exposure) in patients with subsequent PG (outcome).

The current study was grounded on the computerized database of Clalit Health Services (CHS). Ensuring 4.5 million enrollees as of October 2018, CHS is the largest health care organization in Israel, providing healthcare services for 57% of the general Israeli population. The characteristics of the utilized dataset are further detailed in our previous publications [9, 10]. The current study was approved by the institutional review board (IRB) of Ben-Gurion University in accordance with the Declaration of Helsinki.

### Study population

By a systematic check of the dataset of CHS, all individuals with a diagnosis of PG between the years 2000 and 2018 were identified. Subsequently, cases were checked, and only those fulfilling one of the following eligibility criteria were finally eligible: (i) a documented diagnosis of PG registered at least twice by a community board-certified dermatologist, and/or (ii) documentation of the diagnosis of PG in discharge letters of patients admitted to dermatological wards.

All renal comorbidities in the chronic registry of CHS were evaluated in the current study. These are chronic renal failure (CRF), dialysis, kidney transplantation (KT), and other kidney diseases (OKD). OKD was defined based on the diagnostic codes detailed in Supplementary Table 1. Given that these diagnoses were registered in the chronic registry of CHS, they were based on registration by a board-certified nephrologist, suggestive laboratory and imaging data, and were eventually authenticated by the managing general healthcare provider.

**Table 1:** Descriptive characteristics of the study population

Characteristic	Patients with pyoderma gangrenosum (*N*=302)	Controls (*N*=1497)	*P* value
Age, years			
Mean ± SD	54.0 ±20.8	54.0±20.8	1.000
Median (range)	55.8 (0.2–95.1)	55.9 (0.2–95.6)	
Male sex, *N* (%)	157 (57.9%)	629 (58.0%)	0.974
Ethnicity, *N* (%)			
Jews	255 (84.4%)	1264 (84.4%)	1.000
Arabs	47 (15.6%)	233 (15.6%)	
BMI, mg/kg^2^			
Mean ± SD	28.0 ± 6.3	27.8 ± 6.2	0.614
Smoking, *N* (%)	115 (38.1%)	521 (34.8%)	0.274
Charlson comorbidity score			
Mean score ±SD	2.3±2.7	1.3±1.8	<0.001
None (0)	111 (36.8%)	777 (51.9%)	<0.001
Moderate (1–2)	78 (25.8%)	432 (28.9%)	0.276
Severe (≥3)	113 (37.4%)	288 (19.2%)	<0.001

*PG* pyoderma gangrenosum, *N* number, *SD* standard deviation, *BMI* body mass index

The control group included up to 5 control individuals per patient, matched randomly by age, sex, and ethnicity. Prior to their recruitment, control subjects were confirmed to be alive and to contribute longitudinal data for the CHS dataset.

### Main covariates of the study

The study outcome measures were controlled for underlying comorbidities as evaluated by the Charlson comorbidity index (CCI), a validated epidemiological method of quantifying comorbidities. This index has been found to be reliable in predicting mortality and is widely utilized in epidemiological studies [2]. Smoking status was classified as a current smoker or never/past smoker.

### Statistical analysis

Baseline characteristics were described by means and standard deviations (SDs) for continuous variables, while categorical values were signified by percentages. A comparison of sociodemographic and clinical factors between cases and controls was performed using the chi-square test and *t*-test for categorical and continuous variables, respectively.

In the cohort study design, incidence rates of renal comorbidities were calculated for both PG patients and controls and expressed as the number of events per 1000 person-years. Hazard ratios (HRs) for the risk of incident renal comorbidities were obtained by the use of the Cox regression model. In the case-control study design, logistic regression was used to calculate odds ratios (ORs) and 95% confidence intervals (CIs) to compare cases and controls regarding the presence of preceding renal comorbidities. The association was calculated based on individuals who developed PG after the diagnosis of each renal disease, given that a temporal relationship exists between exposure and outcome in case-control studies. Two-tailed *P* values less than 0.05 were considered statistically significant. All statistical analyses were performed using the SPSS software, version 25 (SPSS, Armonk, NY: IBM Corp).

## Results

### Characteristics of the study population

The study population included 302 patients with PG and 1497 age-, sex-, and ethnicity-matched control individuals. The mean (SD) age of the study participants was 54.0 (20.8) years, 57.9% of them were females, and 84.4% were of Jewish ethnicity. Case and control groups were comparable with regard to mean body mass index (BMI) and the prevalence of smoking (Table 1). The mean (SD) CCI score was greater in cases than in controls (2.3 [2.7] vs. 1.3 [1.8], respectively; *P*<0.001). The prevalence of severe comorbid conditions was greater among cases relative to controls (37.4% vs. 19.2%, respectively; *P*<0.001). The characteristics of the study population are outlined in Table 1.

### The risk of PG among individuals with a history of renal disease

A case-control study design was followed to estimate the prevalence of preexisting renal diseases among patients with PG and control individuals. The latter reflects the risk of developing subsequent PG among those with a history of renal diseases. The subsequent development of PG was significantly associated with a history of CRF (OR, 2.04; 95% CI, 1.20–3.47), KT (OR, 5.01; 95% CI, 1.01–24.94), and OKD (OR, 2.04; 95% CI, 1.20–3.47). In a sex-stratified analysis, PG was significantly associated with CRF in both sexes, whereas the association of PG with KT and OKD retained its statistical significance only among females (Table 2).

**Table 2:** The risk of pyoderma gangrenosum among patients with a preexisting history of renal diseases (case-control study design)

Disease	OR (95%CI)	Univariate *P* value	Male-specific OR(95%CI)	Female-specific OR(95%CI)	Adjusted OR (95%CI)^a^	Multivariate *P* value
Chronic renal failure	**2.04 (1.20–3.47)**	**0.007**	1.63 (0.75–3.53)	**2.56 (1.23–5.33)**	**2.34 (1.33–4.11)**	**0.003**
Dialysis	3.81 (0.85–17.10)	0.061	5.15 (0.32–82.86)	3.35 (0.56–20.19)	3.79 (0.84–17.15)	0.084
Kidney transplantation	**5.01 (1.01–24.94)**	**0.029**	**NA**	3.35 (0.55–20.19**)**	**5.03 (1.01–25.12)**	**0.049**
Other kidney diseases	**1.65 (1.03–2.67)**	**0.037**	1.16 (0.55–2.46)	**2.22 (1.18–4.15)**	**1.69 (1.04–2.74)**	**0.033**

*PG* pyoderma gangrenosum, *N* number, *OR* odds ratio, *CI* confidence interval. Bold: significant value

^*^The prevalence of the four diseases in cases when they preceded PG (in cases) or preceded recruitment (in controls)

^a^Multivariate logistic regression model adjusting for age, sex, ethnicity, and comorbidities (as defined by the Charlson comorbidity index)

We then performed a multivariable logistic regression analysis adjusting for putative confounding variables. The latter demonstrated an independently increased risk of PG in individuals with a history of CRF (adjusted OR, 2.34; 95% CI, 1.33–4.11), KT (adjusted OR, 5.03; 95% CI, 1.01–25.12), and OKD (adjusted OR, 1.69; 95% CI, 1.04–2.74), but not in those with a history of dialysis (adjusted OR, 3.79; 95% CI, 0.84–17.15; Table 2).

### The risk of renal diseases among individuals with PG

A retrospective cohort study was performed to disclose the risk of developing renal diseases among patients with PG. The incidence rate of CRF, dialysis, KT, and OKD among patient with PG was 20.0 (95% CI, 13.7–28.5), 3.19 (95% CI, 1.17–7.08), 0.63 (95% CI, 0.03–3.14), and 28.1 (95% CI, 20.4–37.9)/1000 person-year, respectively (Table 3).

**Table 3:** The risk of renal diseases among patients with pyoderma gangrenosum (retrospective cohort study design)

Disease	Chronic renal failure		Dialysis		Kidney transplantation		Other kidney diseases	
	PG	Controls	PG	Controls	PG	Controls	PG	Controls
Follow-up time, PY	1444.92	8179.8	1566.5	8469.4	1572.1	8469.1	1422.5	8013.5
Median follow-up time, years (range)	4.5 (0.0–17.9)	5.5 (0.1–17.8)	4.8 (0.0–17.8)	5.5 (0.0–17.8)	4.8 (0.1–17.8)	5.5 (0.0–17.8)	4.7 (0.0–17.8)	5.4 (0.0–17.8)
Number of events	29	48	5	1	1	1	40	18
Incidence rate / 1000 PY (95% CI)	20.0 (13.7–28.5)	5.9 (4.4–7.7)	3.19 (1.17–7.08)	0.12 (0.06–0.58)	0.63 (0.03–3.14)	0.12 (0.01–0.58)	28.1 (20.4–37.9)	2.25 (1.37–3.48)
Crude HR (95% CI)	**3.19 (1.97–5.16)**	Reference	**26.90 (3.14–230.32)**	Reference	6.07 (0.38–97.41)	Reference	**2.58 (1.48–4.52)**	Reference
Male-specific crude HR (95% CI)	**2.67 (1.30–5.48)**	Reference	NA–	Reference	NA	Reference	**3.38 (1.55–7.39)**	Reference
Female-specific crude HR (95% CI)	**3.70 (1.93–7.10)**	Reference	**5.51 (0.34–88.01)**	Reference	5.54 (0.35–88.50)	Reference	1.99 (0.89 –4.48)	Reference
Adjusted HR (95% CI)^a^	**3.68 (2.72–5.97)**	Reference	**27.79 (3.24–238.14)**	Reference	5.80 (0.36–94.18)	Reference	**2.71 (1.55–4.74)**	Reference

*PG* pyoderma gangrenosum, *N* number, *PY* person-year, *HR* hazard ratio, *CI* confidence interval. Bold: significant value

^a^Multivariate logistic regression model adjusting for age, sex, ethnicity, and comorbidities (as defined by the Charlson comorbidity index)

A significantly increased risk of CRF (HR, 3.19; 95% CI, 1.97–5.16), dialysis (HR, 26.90; 95% CI, 3.14–230.32), and OKD (HR, 2.58; 95% CI, 1.48–4.52) was observed among patients with PG. While the risk of CRF and dialysis was more prominent among females, males were more predisposed to OKD (Table 3). When adjustment for putative confounding factors was performed, patients with PG were found to have an independently elevated risk of CRF (adjusted HR, 3.68; 95% CI, 2.72–5.97), dialysis (adjusted HR, 27.79; 95% CI, 3.24–238.14), and OKD (adjusted HR, 2.71; 95% CI, 1.55–4.74; Table 3).

### Features of PG patients with renal diseases relative to the remaining patients with PG

Table 4 delineates the differential demographic and epidemiological features of patients with PG and coexistent renal comorbidities as compared to the remaining patients with PG. It was invariably shown that patients with a comorbid renal disease presented with PG at a significantly higher age and had higher CCI (Table 4). Patients with PG and coexistent CRF had a more prominent Jewish ethnicity, whereas patients with PG and dialysis had an increased BMI (Table 4).

**Table 4:** Comparison between patients with pyoderma gangrenosum and renal comorbidities relative to the remaining patients with pyoderma gangrenosum

	PG w/ CRF (*n*=49)	PG w/o CRF (*n*=253)	*P* value	PG w/ dialysis (*n*=8)	PG w/o dialysis (*n*=294)	*P* value	PG w/ KT (*n*=4)	PG w/o KT (*n*=298)	*P* value	PG w/ OKD (*n*=42)	PG w/o OKD (n=260‬)‬‬‬‬‬‬‬‬‬‬‬‬‬‬‬‬‬‬‬‬‬‬‬‬‬‬‬‬‬‬‬‬‬‬‬‬‬‬‬‬‬‬‬‬‬‬‬‬‬‬‬‬‬‬‬‬‬‬‬‬‬‬‬‬‬‬‬‬‬‬‬‬‬‬‬‬‬‬‬‬‬‬‬‬‬‬‬‬‬‬‬‬	*P* value
Age at PG onset; years, mean (SD)	68.9 (14.7)	51.2 (20.7)	**<0.001**	59.4 (5.0)	53.9 (21.1)	**0.023**	60.6 (1.9)	54.0 (21.0)	**<0.001**	61.6 (18.1)	52.8 (21,0)	**0.012**
Female sex, *n* (%)	28 (57.1%)	147 (58.1)	0.901	3 (37.5%)	172 (58.5%)	0.235	3 (75.0%)	172 (57.7%)	0.487	23 (54.8%)	152 (58.5)	0.652
Jewish ethnicity, *n* (%)	46 (93.9%)	209 (82.6%)	**0.046**	8 (100.0%)	247 (84.0%)	0.218	4 (100.0%)	251 (84.2%)	0.387	38 (90.5%)	217 (83.5%)	0.245
BMI; Kg/m^2^, mean (SD)	29.4 (6.2)	27.7 (6.2)	0.082	32.9 (6.7)	27.8 (6.2)	**0.025**	31.5 (10.0)	27.9 (6.2)	0.253	29.4 (6.1)	27.7 (6.3)	0.135
Smokers, *n* (%)	20 (40.8%)	95 (37.5%)	0.666	5 (62.5%)	110 (37.4%)	0.149	2 (50.0%)	113 (37.9%)	0.621	19 (45.2%)	96 (36.9%)	0.303
Charlson comorbidity score; mean ±SD	6.1 (2.6)	1.6 (2.0)	**<0.001**	5.4 (1.6)	2.2 (2.7)	**0.001**	3.5 (0.6)	2.3 (2.7)	**0.015**	4.2 (2.9)	2.0 (2.5)	**<0.001**

*PG* pyoderma gangrenosum, *CRF* chronic renal failure, *KT* kidney transplantation, *OKD* other kidney diseases, *n* number, *BMI* body mass index, *w/* with, *w/o* without. Bold: significant value

## Discussion

The current study indicates an increased risk of CRF, dialysis, and OKD among patients with PG relative to controls. Moreover, the presence of a medical history of CRF, KT, and OKD was found to predispose individuals to develop subsequent PG. Relative to the remaining patients with PG, those with PG and chronic renal comorbidities presented with PG at an older age and had an increased burden of comorbid conditions.

The risk of renal comorbidities among patients with PG was not previously explored by controlled observational studies. However, our results, signifying an increased risk of the former, are substantiated by previous case reports of renal involvement in PG manifesting as kidney lesion [11], increase in serum creatinine [12], microhematuria [13], glomerulonephritis [14], aseptic leukocyturia, oliguria, and proteinuria [15]. In a German retrospective, multi-center study encompassing 259 patients with PG, 6.1% of the study participants had renal failure [12].

The increased risk of PG in patients with preexisting renal disease observed in our analysis has also been reported in several case reports. The most frequent renal conditions that were implicated in eliciting PG were KT [16–18], end-stage renal disease (ESRD) [19–21] CRF [22], and renal carcinoma [23].

The pathomechanism underlying the association between PG and renal comorbidities is still unclear. However, several hypotheses have been postulated to account for it. As ulcerative PG oftentimes occurs following minor injuries or surgical operations, a phenomenon coined as pathergy [4], some authors suggested that vascular insufficiency might be involved in the pathogenesis of PG. The latter may be of relevance in the context of PG following radio-cephalic arteriovenous fistula for dialytic treatment [19]. Other authors proposed that the presence of monoclonal gammopathy, which is frequently implicated in triggering PG [3, 4], might lead to a rapid progression of renal failure [14], thus accounting, at least in part, for the observed association. Our outcome measures persisted following the adjustment for CCI which encompasses, among others, a diagnosis of hematological malignancies (leukemia and lymphoma). This multivariate analysis denotes an independent association between PG and renal comorbidities.

This study is epitomized by several strengths such as the population-based setting, the long follow-up period, the relatively large sample of PG patients, the access to all levels of healthcare facilities, and the systematic collection of data. However, a number of limitations in the current study should be acknowledged. The retrospective nature of this study and the use of diagnostic codes limited the ability to collect standardized information on the cutaneous manifestation and severity of the cutaneous disease. To elaborate, differentiation between the ulcerative, vegetative, and atypical subtypes of PG was not feasible using the current dataset. In addition, data regarding exposure to different therapeutic agents could not be retrieved for the current study. The latter is of significance given that cyclosporine, a frequently utilized drug in PG, is implicated with an increased risk of nephrotoxicity [24]. While the likelihood of residual confounding could not be thoroughly refuted, we do not think it had conferred a meaningful impact on the study’s main findings.

To conclude, the current retrospective cohort study disclosed that patients with PG experience an increased risk of developing CRF, dialysis, and OKD. The presence of preexisting CRF, KT, and OKD was associated with the subsequent emergence of PG. Patients with comorbid PG and renal comorbidities present with PG at an older age and have a higher comorbidity score. Further prospective studies are necessary to better characterize the influence of renal impairment on PG and vice versa. A more in-depth investigation may shed light on the underlying pathophysiology of the association between this debilitating dermatologic dermatosis and renal comorbidities.

## Supplementary Information

Below is the link to the electronic supplementary material.

Supplementary file1 (DOCX 23 KB)

## References

[1] Ahronowitz I, Harp J, Shinkai K (2012). Etiology and management of pyoderma gangrenosum: a comprehensive review. Am J ClinDermatol.

[2] Monari P, Moro R, Motolese A, Misciali C, Baraldi C, Fanti PA (2018). Epidemiology of pyoderma gangrenosum: results from an Italian prospective multicentre study. Int Wound J.

[3] Langan SM, Groves RW, Card TR, Gulliford MC. Incidence, mortality, and disease associations of pyoderma gangrenosum in the United Kingdom: a retrospective cohort study. J Invest Dermatol. 2012;132:2166–70. Available from: http://www.ncbi.nlm.nih.gov/pubmed/22534879 10.1038/jid.2012.130 22534879

[4] Kridin K, Cohen AD, Amber KT. Underlying Systemic Diseases in Pyoderma Gangrenosum: A Systematic Review and Meta-Analysis. Am J Clin Dermatol. 2018 [cited 2018 May 13];19:479–87. Available from: http://www.ncbi.nlm.nih.gov/pubmed/29721816 10.1007/s40257-018-0356-7 29721816

[5] Reichrath J, Bens G, Bonowitz A, Tilgen W. Treatment recommendations for pyoderma gangrenosum: An evidence-based review of the literature based on more than 350 patients. J Am Acad Dermatol. 2005;53(2):273–83. 10.1016/j.jaad.2004.10.006. 10.1016/j.jaad.2004.10.006 16021123

[6] Hasselmann DO, Bens G, Tilgen W, Reichrath J. Pyoderma gangrenosum: clinical presentation and outcome in 18 cases and review of the literature. J Dtsch Dermatol Ges [Internet]. 2007;5:560–4. Available from: http://www.ncbi.nlm.nih.gov/pubmed/17610605 10.1111/j.1610-0387.2007.0328.x 17610605

[7] Borda LJ, Wong LL, Marzano AV., Ortega-Loayza AG. Extracutaneous involvement of pyoderma gangrenosum. Arch Dermatol Res. 2019;311(6):425–434. 10.1007/s00403-019-01912-1. 10.1007/s00403-019-01912-1 30923901

[8] Wollina U (2015). Pyoderma gangrenosum-a systemic disease?. Clin Dermatol.

[9] Kridin K, Ludwig RJ, Tzur Bitan D, Kridin M, Damiani G, Cohen AD (2020). Is gout associated with pyoderma gangrenosum? A population-based case-control study. J Clin Med.

[10] Kridin K, Zelber-Sagi S, Comaneshter D, Cohen AD. Coexistent solid malignancies in pemphigus a population-based study. JAMA Dermatol. 2018 [cited 2018 Apr 2];154:435–40. Available from: http://www.ncbi.nlm.nih.gov/pubmed/29453868 10.1001/jamadermatol.2017.6334 PMC5876857 29453868

[11] de Carvalho LR, Zanuncio VV, Gontijo B (2013). Manifestação esplênica e renal do piodermagangrenoso - Relato de caso. An Bras Dermatol.

[12] Al Ghazal P, Herberger K, Schaller J, Strölin A, Hoff N-P, Goerge T, et al. Associated factors and comorbidities in patients with pyoderma gangrenosum in Germany: a retrospective multicentric analysis in 259 patients. Orphanet J Rare Dis. 2013;8:136. Available from: http://www.pubmedcentral.nih.gov/articlerender.fcgi?artid=3844435&tool=pmcentrez&rendertype=abstract. 10.1186/1750-1172-8-136 PMC3844435 24010984

[13] Homsi Y, Pathiparampil J (2018) Pyoderma gangrenosum associated with IgA nephropathy. rheumatology (Sunnyvale) 8: 245. doi: 10.4172/2161-1149.1000245 - Google Search.

[14] Akatsuka T, Kawata T, Hashimoto S, Nakamura S, Koike T. Rapidly progressive renal failure occurring in the course of pyoderma gangrenosum and IgA (λ) monoclonal gammopathy. Intern Med (Japanese Society of Internal Medicine); 1997;36:40–3. 10.2169/internalmedicine.36.40 9058099

[15] Marzano AV, Ishak RS, Lazzari R, Polloni I, Vettoretti S, Crosti C. Vulvar pyoderma gangrenosum with renal involvement. Eur J Dermatol. 2012;22:537–9. 10.1684/ejd.2012.1776 22652578

[16] Alimagham M, Amini-Afshar S, Farahmand S, Pour-Kazemi A, Pour-Reza-Gholi F, Masood S. Frequency of infectious skin lesions in kidney transplant recipients. Urol J [Internet]. 2005;2:193–6. Available from: http://www.ncbi.nlm.nih.gov/pubmed/17602428 17602428

[17] Jha PK, Rana A, Kapoor S, Kher V. Pyoderma gangrenosum in a renal transplant recipient: a case report and review of literature. Indian J Nephrol. 2015;25:297–9. 10.4103/0971-4065.156900 PMC4588326 26628796

[18] Al-Hwiesh AK (2006). Pyoderma gangrenosum in a renal transplant recipient: a case report. Saudsi J Kidney Dis Transpl.

[19] Castillo RF, Cañadas-De La Fuente GA, Husein-Elahmed H, Cantero-Hinojosa J (2011). Pyodermagangrenosum developing over an arteriovenous fistula scar. Intern Med J.

[20] Mohamed Hamzi A, Bahadi A, Alayoud A, Kabbaj D El, Benyahia M. Skin ulcerations in a lupus hemodialysis patient with hepatitis c infection what is your diagnosis? Iran J Kidney Dis; 2013:p. 191. 23689149

[21] Sangiray H, Nguyen JC, Turiansky GW, Norwood CW. Pyoderma gangrenosum occurring near an arteriovenous dialysis shunt. Int. J. Dermatol. John Wiley & Sons, Ltd; 2006;45:851–3. 10.1111/j.1365-4632.2006.02484.x 16863525

[22] Goto M, Okamoto O, Fujiwara S, Yanagi T, Komada S, Yokoyama S (2002). Vegetative pyoderma gangrenosum in chronic renal failure. Br J Dermatol.

[23] Regnier-Rosencher E, Bizet N, Méry L. Pyoderma gangrenosum associated with renal carcinoma. J. Am. Acad. Dermatol. Mosby Inc.; 2011;64:1208–11. 10.1016/j.jaad.2009.09.039 21571198

[24] Busauschina A, Schnuelle P, Van Der Woude FJ (2004). Cyclosporine nephrotoxicity. Transplant Proc.

