# Supplementary material for: Chronic renal comorbidities in pyoderma gangrenosum: a retrospective cohort study
Source: Immunol Res. 2021 Apr 22;69(3):249–54. doi: 10.1007/s12026-021-09187-3 (PMC8266709; doi:10.1007/s12026-021-09187-3)
Supplement: Supplementary file 1 — Supplementary file1 (DOCX 23 KB) [file 12026_2021_9187_MOESM1_ESM.docx]

**Supplementary table 1-** Diagnostic codes constituting the diagnosis of ‘other kidney diseases’

| **ICD9 Code** | **ICD9 Name** |
| --- | --- |
| 2741 | GOUTY NEPHROPATHY |
| 27410 | GOUTY NEPHROPATHY, UNSPECIFIED |
| 27411 | URIC ACID NEPHROLITHIASIS |
| 27419 | OTHER GOUTY NEPHROPATHY |
| 4401 | ATHEROSCLEROSIS OF RENAL ARTERY |
| 581 | NEPHROTIC SYNDROME |
| 5810 | NEPHROTIC SYN.LESION OF PROLIFERATIVE GLOMERULONEPHRITIS |
| 5811 | NEPHROTIC SYN.WITH MEMBRANOUS GLOMERULONRPHRITIS |
| 5812 | NEPHROTIC SYN.WITH MEMBRANOPROLIFERATIVE GLOMERULONEPHRITIS |
| 5813 | NEPHROTIC SYN.WITH LESION OF MINIMAL CHANGE GLOMERULONEPHRITIS |
| 5818 | NEPHROTIC SYN.WITH OTHER SPEC.PATHOLOGICAL LESION IN KIDNEY |
| 58181 | NEPHROTIC SYNDROME IN DISEASES CLASSIFIED ELSEWHERE |
| 58189 | NEPHROTIC SYN.WITH OTHER SPEC.PATHOLOGICAL LESION IN KIDNEY |
| 5819 | NEPHROTIC SYNDROME WITH UNSP.PATHOLOGICAL LESION IN KIDNEY |
| 582 | CHRONIC GLOMERULONEPHRITIS |
| 5820 | CHR.GLOMERULONEPHRITIS WITH PROLIFERATIVE GLOMERULONEPHR. |
| 5821 | CHR.GLOMERULONEPHRITIS WITH MEMBRANOUS GLOMERULONEPHRITIS |
| 5822 | CHR.GLOMERULONEPHRITIS WITH MEMBRANOPROLIF.GLOMERULONEPHR. |
| 5824 | CHR.GLOMERULONEPHRITIS WITH RAPIDLY PROGRESS.GLOMERULONEPHRITIS |
| 5828 | CHR.GLOMERULONEPHRITIS WITH OTHER SPEC.PATH.LESION IN KIDNEY |
| 58281 | CHRONIC GLOMERULONEPHRITIS IN DISEASES CLASSIFIED ELSEWHERE |
| 58289 | CHR.GLOMERULONEPHRITIS WITH OTHER SPEC.PATH.LESION IN KIDNEY |
| 5829 | CHR.GLOMERULONEPHRITIS WITH UNSP.PATHOL.LESION IN KIDNEY |
| 583 | NEPHRITIS AND NEPHROPATHY, NOT SPECIFIED AS ACUTE OR CHRONIC |
| 5830 | NEPHRITIS AC.OR CHR.,WITH PROLIFER.GLOMERULONEPHRITIS |
| 5831 | NEPHRITIS AC.OR CHR.,WITH MEMBRAN.GLOEMRULONEPHRITIS |
| 5832 | NEPHRITIS AC.OR CHR.,WITH MEMBRANOPROLIF.GLOMERULONEPHRITIS |
| 5834 | NEPHRITIS AC.OR CHR.,WITH RAPID.PROGR.GLOMERULONEPHRITIS |
| 5836 | NEPHRITIS AC.OR CHR.,WITH RENAL CORTICAL NECROSIS |
| 5837 | NEPHRITIS AC.OR CHR.,WITH RENAL MEDULLARY NECROSIS |
| 5838 | NEPHRITIS AC.OR CHR.WITH OTHER SPEC.LESIONS IN KIDNEY |
| 58381 | NEPHRITIS AC.OR CHR.,IN DISEASES CLASSIFIED ELSEWHERE |
| 58389 | NEPHRITIS,AC.OR CHR.WITH OTHER SPEC.PATH.LESIONS IN KIDNEY |
| 5839 | NEPHRITIS/NEPHROPATHY,ACUTE OR CHRONIC;GLOMERULONEPHRITIS NOS. |
| 587 | RENAL SCLEROSIS, UNSPECIFIED |
| 588 | DISORDERS RESULTING FROM IMPAIRED RENAL FUNCTION |
| 5880 | RENAL OSTEODYSTROPHY |
| 5881 | NEPHROGENIC DIABETES INSIPIDUS |
| 5888 | SPECIFIED DISORDERS RESULTING FROM IMPAIRED RENAL FUNCTION |
| 5889 | UNSP.DISORDER RESULTING FROM IMPAIRED RENAL FUNCTION |
| 589 | SMALL KIDNEY OF UNKNOWN CAUSE |
| 5890 | UNILATERAL SMALL KIDNEY |
| 5891 | BILATERAL SMALL KIDNEYS |
| 5899 | SMALL KIDNEY, UNSPECIFIED |
| 5900 | CHRONIC PYELONEPHRITIS |
| 59000 | CHRON.PYELONEPHRITIS WITHOUT LESION OF RENAL MEDULL.NECROSIS |
| 59001 | CHRONIC PYELONEPHRITIS WITH LESION OF RENAL MEDULLARY NECROSIS |
| 5903 | PYELOURETERITIS CYSTICA |
| 5908 | PYELONEPHRITIS OR PYONEPHROSIS,NOT SPEC.AS ACUTE OR CHR. |
| 59080 | PYELONEPHRITIS, UNSPECIFIED |
| 59081 | PYELITIS OR PYELONEPHRITIS IN DISEASES CLASSIFIED ELSEWHERE |
| 5909 | INFECTION OF KIDNEY, UNSPECIFIED |
| 591 | HYDRONEPHROSIS |
| 592 | CALCULUS OF KIDNEY AND URETER |
| 5920 | CALCULUS OF KIDNEY |
| 5921 | CALCULUS OF URETER |
| 5929 | URINARY CALCULUS, UNSPECIFIED |
| 593 | OTHER DISORDERS OF KIDNEY AND URETER |
| 5930 | NEPHROPTOSIS |
| 5931 | HYPERTROPHY OF KIDNEY |
| 5932 | CYST OF KIDNEY, ACQUIRED |
| 5933 | STRICTURE OR KINKING OF URETER |
| 5934 | OTHER URETERIC OBSTRUCTION |
| 5935 | HYDROURETER |
| 5936 | POSTURAL PROTEINURIA |
| 5937 | VESICOURETERAL REFLUX |
| 59370 | VESICOURETERAL REFLUX, UNSP. OR WITHOUT REFLUX NEPHROPATHY |
| 59371 | VESICOURETERAL REFLUX WITH REFLUX NEPHROPATHY, UNILATERAL |
| 59372 | VESICOURETERAL REFLUX WITH REFLUX NEPHROPATHY, BILATERAL |
| 59373 | VESICOURETERAL REFLUX WITH REFLUX NEPHROPATHY, NOS |
| 5938 | OTHER SPECIFIED DISORDERS OF KIDNEY AND URETER |
| 59381 | VASCULAR DISORDERS OF KIDNEY |
| 59382 | URETERAL FISTULA |
| 59389 | OTHER SPECIFIED DISORDERS OF KIDNEY AND URETER |
| 593890 | DILATEATION OF COLLECTING SYSTEM |
| 593891 | DILATATION OF RENAL PELVIC (PYELECTASIA) |
| 5939 | UNSPECIFIED DISORDER OF KIDNEY AND URETER |
| 596 | OTHER DISORDERS OF BLADDER |
| 5960 | BLADDER NECK OBSTRUCTION |
| 5961 | INTESTINOVESICAL FISTULA |
| 5962 | VESICAL FISTULA, NOT ELSEWHERE CLASSIFIED |
| 5963 | DIVERTICULUM OF BLADDER |
| 5964 | ATONY OF BLADDER |
| 5965 | OTHER FUNCTIONAL DISORDERS OF BLADDER |
| 59651 | HYPERTONICITY (HYPERACTIVITY) OF BLADDER |
| 59652 | LOW BLADDER COMPLIANCE |
| 59653 | PARALYSIS OF BLADDER |
| 59654 | NEUROGENIC BLADDER NOS |
| 59655 | DETRUSOR SPHINCTER DYSSYNERGIA |
| 59659 | OTHER FUNCTIONAL DISORDER OF BLADDER (DETRUSOR INSTABILITY) |
| 5966 | RUPTURE OF BLADDER, NONTRAUMATIC |
| 5967 | HEMORRHAGE INTO BLADDER WALL |
| 5968 | OTHER SPECIFIED DISORDERS OF BLADDER |
| 5969 | UNSPECIFIED DISORDER OF BLADDER |
| 598 | URETHRAL STRICTURE |
| 5980 | URETHRAL STRICTURE DUE TO INFECTION |
| 59800 | URETHRAL STRUCTURE DUE TO UNSPECIFIED INFECTION |
| 59801 | URETHRAL STRUCTURE DUE TO INFECTIVE DISEASES CLASS.ELSEWHERE |
| 5981 | TRAUMATIC URETHRAL STRICTURE |
| 5982 | POSTOPERATIVE URETHRAL STRICTURE |
| 5988 | OTHER SPECIFIED CAUSES OF URETHRAL STRICTURE |
| 5989 | URETHRAL STRICTURE, UNSPECIFIED |
| 753 | CONGENITAL ANOMALIES OF URINARY SYSTEM |
| 7530 | RENAL AGENESIS AND DYSGENESIS |
| 75301 | POTTERS SYNDROME |
| 75302 | UNILATERAL RENAL AGENESIS |
| 7531 | CONGENITAL CYSTIC KIDNEY DISEASE |
| 75310 | CONGENITAL CYSTIC KIDNEY DISEASE, UNSPECIFIED |
| 75311 | CONGENITAL SINGLE RENAL CYST |
| 75312 | CONGENITAL POLYCYSTIC KIDNEY, UNSPECIFIED TYPE |
| 75313 | CONGENITAL POLYCYSTIC KIDNEY, AUTOSOMAL DOMINANT |
| 75314 | CONGENITAL POLYCYSTIC KIDNEY, AUTOSOMAL RECESSIVE |
| 75315 | CONGENITAL RENAL DYSPLASIA |
| 75316 | CONGENITAL MEDULLARY CYSTIC KIDNEY (NEPHRONOPTHISIS) |
| 75317 | CONGENITAL MEDULLARY SPONGE KIDNEY |
| 75319 | OTHER SPECIFIED CONGENITAL CYSTIC KIDNEY DISEASE |
| 753191 | MULTICYSTIC KIDNEY |
| 7532 | CONGENITAL OBSTRUCTIVE DEFECTS OF RENAL PELVIS AND URETER |
| 75320 | UNSP. OBSTRUCTIVE DEFECT OF RENAL PELVIS & URETER |
| 75321 | CONGENITAL OBSTRUCTION OF URETEROPELVIC JUNCTION |
| 75322 | CONGENITAL OBSTRUCTION OF URETEROVESICAL JUNCTION |
| 753221 | CONGENITAL HYDROURETER |
| 75323 | CONGENITAL URETEROCELE |
| 75329 | OTHER OBSTRUCTIVE DEFECTS OF RENAL PELVIS & URETER |
| 753290 | CONGENITAL HYDRONEPHROSIS |
| 7533 | OTHER SPECIFIED CONGENITAL ANOMALIES OF KIDNEY |
| 75331 | HORSESHOE KIDNEY |
| 75332 | CONGENITAL ECTOPIC KIDNEY |
| 75333 | CONGENITAL PTOTIC KIDNEY |
| 75334 | CONGENITAL ENLARGED (GIANT) KIDNEY |
| 75335 | DOUBLE KIDNEY WITH DOUBLE PELVIS |
| 75336 | EXTRA-RENAL PELVIS |
| 7534 | OTHER SPECIFIED CONGENITAL ANOMALIES OF URETER |
| 75342 | CONGENITAL ECTOPIC URETER |
| 75343 | DOUBLE COLLECTING SYSTEM |
| 7535 | EXSTROPHY OF URINARY BLADDER |
| 7536 | CONGENITAL ATRESIA AND STENOSIS OF URETHRA AND BLADDER NECK |
| 75361 | CONGENITAL STENOSIS OF URINARY MEATUS |
| 7537 | CONGENITAL ANOMALIES OF URACHUS |
| 7538 | OTHER SPECIFIED CONGENITAL ANOMALIES OF BLADDER AND URETHRA |
| 7539 | UNSPECIFIED CONGENITAL ANOMALY OF URINARY SYSTEM |
